# Integrated Metabolomics and Transcriptomics Reveal the Influence of Natural and Cultivation-Managed Habitats on Metabolic Divergence and Flavonoid Enrichment in *Anoectochilus roxburghii*

**DOI:** 10.3390/metabo16050294

**Published:** 2026-04-27

**Authors:** Erli Wang, Weicheng Gao, Peng Wang, Xiaoping Wang

**Affiliations:** 1 School of Pharmacy, Zhangzhou Health Vocational College, Zhangzhou 363000, China; wangel@zzwzy.edu.cn (E.W.);; 2 Zhiran Biotechnology Co., Ltd., Tianjin 301000, China; xiang_nan_1999@163.com

**Keywords:** *Anoectochilus roxburghii*, metabolomics, transcriptomics, flavonoids

## Abstract

**Background/Objectives**: Environmental conditions in natural and cultivation-managed habitats strongly influence plant physiology and medicinal quality. However, the molecular mechanisms underlying metabolic differentiation in *Anoectochilus roxburghii* remain poorly understood. This study aimed to elucidate the metabolic and transcriptional differences between wild and cultivated *A. roxburghii* and to identify the regulatory mechanisms driving habitat-associated variation in metabolite profiles. **Methods**: We applied integrated non-targeted metabolomics and transcriptomics to compare metabolic profiles and gene expression in the leaves and stems of 15-month-old wild and cultivated *A. roxburghii* plants. Gene–metabolite correlation analysis was performed to identify coordinated correlation networks associated with key biosynthetic pathways. **Results**: Our analyses revealed clear differences in metabolite composition and transcriptional patterns between habitat types, suggesting distinct strategies of metabolic resource allocation. Wild plants showed significant enrichment of amino acids and other primary metabolites, whereas cultivated plants accumulated higher levels of flavonoids. Gene–metabolite correlation analysis indicated that multiple flavonoid metabolites were closely associated with key structural genes, including *F3H*, *C12RT1*, and *HHT1*, forming a tightly connected correlation network. In addition, several transcription factor families, including MYB, bHLH, WRKY, and AP2/ERF, showed strong correlations with genes involved in the flavonoid pathway, suggesting that flavonoid accumulation in cultivated plants may be associated with coordinated transcriptional control. **Conclusions**: Taken together, these findings suggest that habitat conditions are associated with differences in metabolic networks and resource allocation in *A. roxburghii*. This work provides new insight into the metabolic plasticity of this medicinal plant and highlights potential factors associated with molecular mechanisms that may contribute to variation in medicinal quality.

## 1. Introduction

*Anoectochilus roxburghii*, a member of the genus *Anoectochilus* in the family Orchidaceae, is a rare medicinal plant widely used in traditional Chinese medicine [1]. Often referred to as the “King of Herbs” or “Black Ginseng,” its therapeutic value is largely attributed to the presence of diverse bioactive secondary metabolites. Previous studies have reported that *A. roxburghii* contain s multiple classes of compounds, including amino acids, flavonoids, polysaccharides, alkaloids, and volatile oils, which contribute to its anti-inflammatory, antioxidant, immunomodulatory, and hypoglycemic activities [2,3,4]. However, extensive harvesting of wild populations [5] has led to a marked decline in natural resources, resulting in the inclusion of *A. roxburghii* in the China Biodiversity Red List (2021). To ensure a stable supply, artificial cultivation has increasingly replaced wild harvesting. In cultivation systems, plants are typically grown under shaded, humid, forest-like conditions, characterized by low light intensity, high humidity, and organic-rich substrates, to mimic their natural understory environment [6]. Despite these efforts, cultivation conditions may alter plant metabolism, raising concerns about whether cultivated plants maintain the same bioactive characteristics as their wild counterparts.

Among the bioactive constituents identified in A. roxburghii, flavonoids are widely considered the main contributors to its pharmacological activity [7]. Phytochemical studies using analytical techniques such as HPLC and UPLC-QTOF-MS have identified more than 50 flavonoid compounds, including vitexin, isovitexin, and apigenin-7-O-glucoside, as well as various flavonoid glycosides and quercetin derivatives [1,8,9]. Previous studies have investigated flavonoid accumulation in *A. roxburghii*, particularly in leaves, under different cultivation conditions [10]. However, comparative analyses of flavonoid metabolites between plant tissues, especially between leaves and stems, remain limited.

In recent years, the integration of metabolomics and transcriptomics has become a powerful approach for studying plant secondary metabolism and elucidating complex biosynthetic pathways in medicinal plants. Metabolomics provides comprehensive profiling of metabolite composition, while transcriptomics reveals gene expression patterns underlying metabolic variation. Combining these approaches enables the identification of gene–metabolite relationships and facilitates reconstruction of metabolic pathways. Studies have shown that flavonoid biosynthesis is regulated by coordinated expression of key structural genes, including phenylalanine ammonia-lyase (*PAL*), chalcone synthase (*CHS*), chalcone isomerase (*CHI*), flavanone 3-hydroxylase (*F3H*), and dihydroflavonol 4-reductase (*DFR*) [11,12,13,14]. These genes are often controlled by transcription factors such as MYB, bHLH, and WD40 proteins, which form conserved regulatory complexes. For example, integrated multi-omics analysis in Panax ginseng has revealed spatiotemporal regulatory networks governing ginsenoside biosynthesis and identified key enzyme genes such as CYP716A53v2 [15]. In contrast, existing transcriptomic studies on *A. roxburghii* have mainly focused on preliminary identification of genes involved in the phenylpropanoid and flavonoid pathways, while metabolomic studies have largely examined selected metabolites using targeted approaches. As a result, the overall metabolic landscape and gene–metabolite relationships underlying flavonoid biosynthesis in *A. roxburghii* remain poorly understood.

To address these gaps, we applied an integrated metabolomics and transcriptomics strategy to systematically compare metabolite profiles and gene expression patterns in the leaves and stems of wild and cultivated *A. roxburghii*. In particular, we investigated whether cultivation leads to tissue-specific metabolic changes, with a focus on flavonoid biosynthesis and its regulatory features. By integrating gene–metabolite associations across tissues and habitats, this study provides a comprehensive framework for understanding cultivation-related metabolic divergence in *A. roxburghii* and offers insights into the molecular basis underlying variation in its medicinal quality.

## 2. Method

### 2.1. Plant Materials, Experimental Design, and Sample Collection

The *A. roxburghii* (Jewel Orchid) plants used in this study were obtained from both a wild population and a forest-understory semi-wild cultivated population in Fujian Province, China. Wild individuals were collected from a natural distribution site in Shajian Town, Hua’an County, Zhangzhou City (24°47′ N, 117°05′ E; elevation 1020 m). This habitat is characterized by a humid ravine environment within an evergreen broad-leaved forest dominated by mixed coniferous and broad-leaved vegetation. The soil consists of a thick humus layer overlying typical yellow-red forest soil, which represents the natural ecological conditions for *A. roxburghii.* Cultivated samples were obtained from a forest-understory semi-wild cultivation base located in Shuyang Town, Nanjing County, Zhangzhou City (24°35′ N, 117°05′ E; elevation 1052 m). Here, plants are grown under natural forest canopy conditions with approximately one-third of full sunlight and an average annual temperature of 20 °C. The cultivation substrate consists of native forest humus soil formed from decomposed leaf litter (approximately 10 cm thick). No chemical fertilizers are applied, and plant nutrition is supplied entirely by natural organic matter in the substrate. Moisture is maintained primarily through natural rainfall and supplemented with periodic misting using mountain spring water.

To reduce variation associated with plant developmental stage, both wild and cultivated plants were sampled during the same season (mid-October) and within the same time window (09:00–11:00). Only healthy adult plants at a stable vegetative stage were selected based on uniform morphological criteria: plant height ≥ 12 cm, 5–6 fully expanded leaves, well-developed root systems, and no visible disease or pest damage. For the cultivated population, plants grown under understory conditions for approximately 15 months were sampled.

A total of 30 cultivated plants were collected, and 10 plants were pooled to form one biological replicate (three replicates in total). For the wild population, 21 individuals were collected and grouped into three biological replicates (seven plants per replicate) using the same selection criteria (Table S1). Pooling multiple individuals per replicate was performed to minimize individual-level variability and to better represent population-level metabolic profiles. Potential batch effects were evaluated by PCA based on metabolomic and transcriptomic datasets. No obvious batch-associated clustering was observed. The two sampling sites are located within the mountainous region of Zhangzhou, Fujian Province, and share similar elevation ranges, subtropical monsoon climate, and forest ecosystem characteristics, thereby reducing environmental differences between the wild and cultivated populations.

All samples were taxonomically identified as *A. roxburghii* by Professor Wang Xiaoping (Zhangzhou Health Vocational College). Voucher specimens were deposited in the institutional herbarium. Fresh tissues were immediately frozen and stored at −80 °C for subsequent metabolomic and transcriptomic analyses.

Leaves and stems were collected separately from both wild and cultivated plants, generating four experimental groups: wild leaves (WL), wild stems (WS), cultivated leaves (CL), and cultivated stems (CS), each with three biological replicates. After collection, surface debris was removed, and tissues were briefly rinsed with deionized water, blotted dry, rapidly frozen in liquid nitrogen within 5 min of harvesting, and stored at −80 °C until metabolomic and transcriptomic analyses.

### 2.2. Metabolomic Data Acquisition and Analysis

Leaf and stem tissues stored at −80 °C were ground into a fine powder under liquid nitrogen. Approximately 50 mg of each sample was transferred into a 2 mL centrifuge tube, and 400 μL of extraction solvent (methanol:water, 4:1, *v*/*v*) containing 0.02 mg/mL L-2-chlorophenylalanine as an internal standard was added. A 6 mm stainless-steel bead was included in each tube, and the samples were homogenized using a grinding mill for 5 min (−10 °C, 50 Hz). The homogenates were subjected to ultrasonic extraction for 30 min at low temperature (5 °C, 40 kHz), followed by incubation at −20 °C for 30 min to promote protein precipitation. The samples were then centrifuged at 13,000× *g* for 15 min at 4 °C, and the resulting supernatants were collected for LC–MS/MS analysis.

A pooled quality control (QC) sample was prepared by mixing equal aliquots from all extracts to evaluate analytical reproducibility. During the LC–MS/MS run, one QC sample was injected after every 5–15 analytical samples to monitor instrument stability and data consistency.

Metabolite profiling was performed using LC-MS/MS on a UHPLC-Q Exactive HF-X system (Thermo Fisher Scientific, Waltham, MA, USA) equipped with an ACQUITY HSS T3 column (100 mm × 2.1 mm; 1.8 μm; Waters, Milford, MA, USA). The analysis was conducted at Majorbio Bio-Pharm Technology Co., Ltd. (Shanghai, China). Raw data were processed using Progenesis QI software (v2.3, Waters, Milford, MA, USA) for peak detection, retention time alignment, and signal normalization. Metabolite identification was achieved by comparison with the HMDB, METLIN, and Majorbio in-house spectral databases. Data preprocessing included filtering features using the 80% rule (signals detected in ≥80% of samples within at least one experimental group). Missing values were replaced with the minimum detected value, and variables with a relative standard deviation (RSD) greater than 30% in the QC samples were excluded. The remaining data were normalized by total signal intensity and log10-transformed prior to statistical analysis.

Multivariate statistical analyses, including principal component analysis (PCA) and orthogonal partial least squares discriminant analysis (OPLS-DA), were performed using the ropls package in R. Differential metabolites were identified based on a variable importance in projection (VIP) score greater than 1 from the OPLS-DA model and a Student’s *t*-test, with *p* < 0.05 [16]. The identified metabolites were classified based on KEGG and HMDB annotations, and KEGG pathway enrichment was conducted using Fisher’s exact test with FDR correction [17,18]. Metabolites associated with amino acid, carbohydrate, lipid, nucleotide, and central energy metabolism were categorized as primary metabolites, whereas those involved in specialized metabolic pathways, including flavonoid, phenylpropanoid, terpenoid, and alkaloid biosynthesis, were classified as secondary metabolites [18,19].

### 2.3. Transcriptomic Data Acquisition and Analysis

Total RNA was extracted using TRIzol reagent and quantified with a NanoDrop 2000 spectrophotometer (Thermo Fisher Scientific, Waltham, MA, USA). RNA integrity was evaluated using an Agilent 5300 system, and only high-quality RNA samples (OD260/280 = 1.8–2.2 and RNA Quality Number (RQN) ≥ 6.5) were used for library preparation [20]. Strand-specific mRNA libraries were constructed using the Illumina Stranded mRNA Prep Ligation kit (Illumina, San Diego, CA, USA) with 1 μg of total RNA as input. Poly(A)+ RNA was enriched using oligo(dT) magnetic beads, fragmented, and reverse-transcribed into cDNA. Subsequent steps included end repair, adapter ligation, size selection to obtain fragments of approximately 300–400 bp, and PCR amplification (10–15 cycles). The libraries were sequenced on an Illumina NovaSeq X Plus platform (Illumina, San Diego, CA, USA) using paired-end 150 bp reads [21].

Because a high-quality reference genome for *A. roxburghii* is currently unavailable, transcriptome reconstruction was performed using a de novo assembly strategy. Raw sequencing reads were first quality-filtered using fastp [22], and the resulting clean reads were assembled using Trinity. Redundant sequences were removed using CD-HIT, and assembly quality was evaluated with TransRate and BUSCO.

Functional annotation of the assembled transcripts was performed by searching against the NR, COG, and KEGG databases using DIAMOND, and Gene Ontology (GO) terms were assigned using Blast2GO. Gene and transcript abundances were quantified with RSEM based on mapped clean reads [23]. Differential gene expression analysis was conducted using DESeq [24], and genes with FDR < 0.05 and |log_2_FC| ≥ 1 were considered differentially expressed genes (DEGs). Functional enrichment analyses for GO terms and KEGG pathways were performed using Fisher’s exact test with FDR correction.

### 2.4. Integrated Analysis of Metabolomics, Transcriptomics, and Transcription Factors

KEGG pathway enrichment analyses for differential metabolites and DEGs were performed separately using Fisher’s exact test with Benjamini–Hochberg FDR correction. Pathways significantly enriched in both datasets were identified as shared pathways and used for integrated multi-omics pathway analysis. For flavonoid-related pathways, Pearson correlation analysis was performed to evaluate relationships between metabolite abundance and gene expression levels. Significant correlations were defined as a Pearson correlation coefficient (*r*) > 0.6 and *p* < 0.05. Based on these relationships, metabolite–gene interaction networks were constructed and visualized using Cytoscape (v3.10.4) [25].

To further investigate regulatory mechanisms underlying metabolic variation, transcription factors (TFs) were identified by annotating the assembled transcripts against the Plant Transcription Factor Database (PlantTFDB) [26]. Putative TFs were classified into families based on conserved domain information, and their expression profiles were examined alongside structural genes involved in flavonoid biosynthesis. This analysis supported the construction of putative regulatory networks linking transcription factors, structural genes, and flavonoid-related metabolites.

## 3. Results

### 3.1. Metabolomic Profiling Reveals Metabolic Differences Between Wild and Cultivated A. roxburghii

After database matching and data preprocessing, a total of 2257 metabolites were detected across all samples in both positive and negative ionization modes, including 1630 detected in the positive mode and 627 in the negative mode. Based on phytochemical classification and functional annotation, these metabolites were grouped into four categories: primary metabolites, secondary metabolites, other metabolites, and unknown compounds (Figure S1). Among the annotated compounds, 457 were classified as primary metabolites, representing 38% of the identified metabolites. These mainly included amino acid derivatives, carbohydrates, lipids, and nucleotides. Secondary metabolites comprised 421 compounds (35%), with the major classes including flavonoids, terpenoids, phenolic acids, and alkaloid-related compounds.

**Figure 1:**
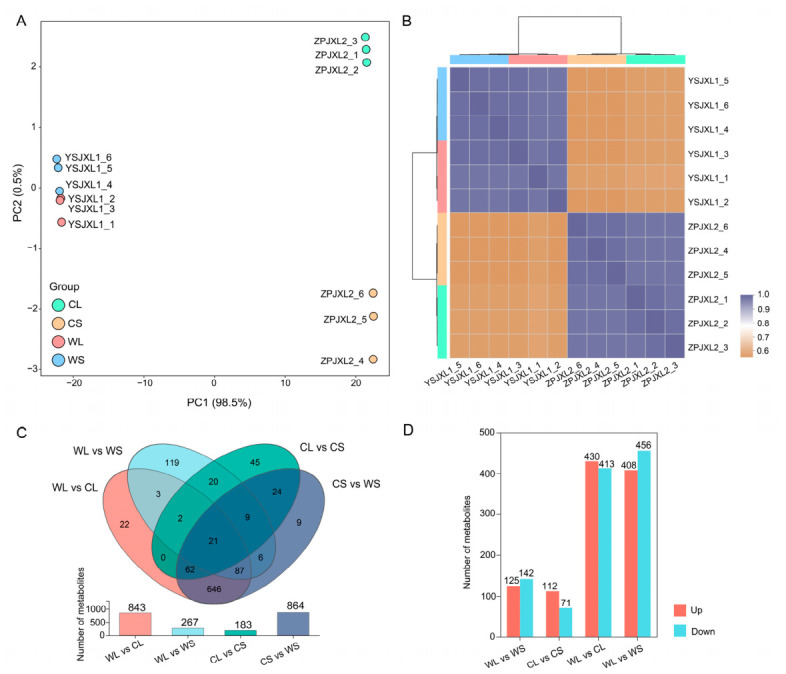
Overview of differential metabolite analysis across sample groups of wild and cultivated *Anoectochilus roxburghii*. (**A**) Principal component analysis of differential metabolites; (**B**) sample correlation heatmap showing relationships among groups; (**C**) Venn diagram illustrating the shared and unique differential metabolites across comparisons; (**D**) numbers of upregulated and downregulated metabolites identified in each comparison group.

To examine overall metabolic variation among samples, PCA was performed for the four groups: WL, WS, CL, and CS. The PCA score plot showed a clear separation between wild and cultivated samples along the first principal component (PC1), indicating distinct metabolic profiles associated with specific habitats (Figure 1A). Along the second principal component (PC2), leaf and stem tissues showed partial separation, suggesting additional tissue-specific metabolic variation. Consistent with this pattern, the sample correlation heatmap showed high similarity within groups and clear clustering between wild and cultivated samples (Figure 1B).

To further identify metabolites responsible for these differences, differential metabolite analysis was conducted using the criteria VIP ≥ 1 and *p* < 0.05. Venn diagram analysis identified 407 differential metabolites shared across all comparisons (Figure 1C). Pairwise comparisons revealed substantial metabolic divergence between habitats, with 843 differential metabolites detected between wild and cultivated leaves (WL vs. CL) and 864 between wild and cultivated stems (WS vs. CS). In contrast, comparisons between tissues of plants from the same habitat showed fewer differences, with 267 differential metabolites between leaves and stems in wild plants (WL vs. WS) and 183 in cultivated plants (CL vs. CS). These results indicate that habitat-associated variation contributes more strongly to metabolic differentiation than tissue-specific differences.

Analysis of the direction of metabolite changes further supported this pattern (Figure 1D). In leaves, 430 metabolites were upregulated and 413 were downregulated in cultivated samples relative to wild samples. Similarly, in stems, 456 metabolites were upregulated and 408 were downregulated. The relatively balanced distribution of up- and downregulated metabolites suggests widespread metabolic reprogramming between wild and cultivated habitats.

### 3.2. Differential Metabolite Patterns in Wild and Cultivated A. roxburghii

**Figure 2:**
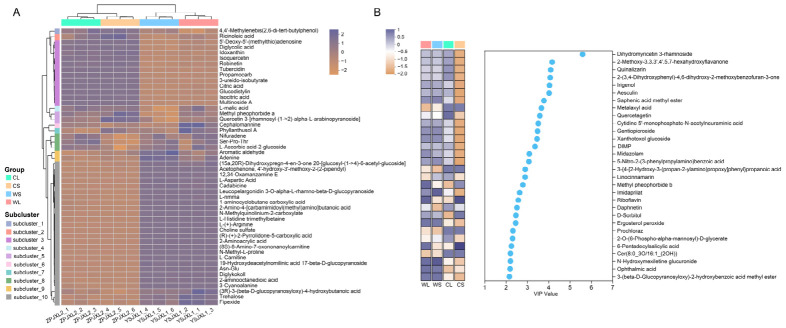
Clustering and variable importance analysis of differential metabolites in wild and cultivated *Anoectochilus roxburghii*. (**A**) Heatmap showing the top 50 differential metabolites across the sample groups; (**B**) VIP scores of the top 30 metabolites derived from the OPLS-DA model.

To further characterize the distribution of differential metabolites and their contribution to sample discrimination, hierarchical clustering and VIP ranking were performed (Figure 2). Heatmap clustering of the top 50 differential metabolites showed clear separation between wild and cultivated *A. roxburghii* samples in both leaf and stem tissues (Figure 2A). Wild plants were characterized by relatively higher levels of several primary metabolites, particularly amino acids and their derivatives, including N-methyl-L-proline, L-histidine trimethylbetaine, 3-cyanoalanine, 2-aminoacrylic acid, L-(+)-arginine, and L-aspartic acid, suggesting shifts in nitrogen-associated metabolic states. Pairwise comparisons further supported this pattern (Figure S2). In both leaf (WL vs. CL) and stem (WS vs. CS) comparisons, several nitrogen-containing metabolites, such as Arg–Ser–Asn and 4′-thiothymidine, were consistently enriched in wild plant tissues. In contrast, cultivated samples showed relatively higher abundances of multiple flavonoids and phenolic compounds, including isoquercetin, robinetin, and dihydromyricetin 3-rhamnoside (Figure 2A). Differential metabolite analysis also indicated that several specialized metabolites, including 7-O-prenylscopoletin, carboxymethyl isoferulate, and other lipid- and phenolic-related compounds, accumulated at higher levels in cultivated plants across pairwise comparisons (Figure S2).

Consistent with these patterns, multivariate PLS-DA analysis showed that metabolites with the highest VIP scores were predominantly flavonoids and phenolic derivatives (Figure 2B). This suggests that although primary metabolic changes are prominent in wild plants, secondary metabolites, particularly flavonoids and phenolics, play a major role in distinguishing wild and cultivated populations [27].

Tissue-specific metabolic differences were examined through pairwise comparisons within each habitat (WL vs. WS and CL vs. CS) (Figure S2). Under wild conditions, metabolic differences between leaves and stems were relatively moderate. WL showed higher levels of several nitrogen-containing and peptide-related metabolites, including Arg–Ser–Asn, ophthalmic acid, and 4′-thiothymidine, whereas WS exhibited increased abundance of sugar- and nucleotide-related metabolites such as 4,6-O-ethylidene-D-glucose and orotidine, along with several terpenoid-like compounds. In cultivated plants, tissue differentiation was more pronounced. Cultivated leaves (CL) displayed higher levels of metabolites such as 7-O-prenylscopoletin and agmatine, whereas cultivated stems (CS) were enriched in multiple flavonoid- and phenolic-related compounds, including gossypin, gossypetin, 2,2′,3,3′-tetrahydroxystilbene, and chromen-4-one derivatives. In addition, antioxidant-associated metabolites, such as L-glutathione and γ-Glu–Cys, together with lipid-related compounds, including phosphatidylglycerol (PG)-associated metabolites, were relatively enriched in cultivated stems.

Overall, these results indicate that tissue-associated metabolic variation depends on habitat context. Compared with wild plants, cultivated *A. roxburghii* plants exhibit stronger tissue-specific differentiation, particularly in secondary metabolite profiles.

### 3.3. Metabolic Pathway Enrichment of Differential Metabolites in A. roxburghii

**Figure 3:**
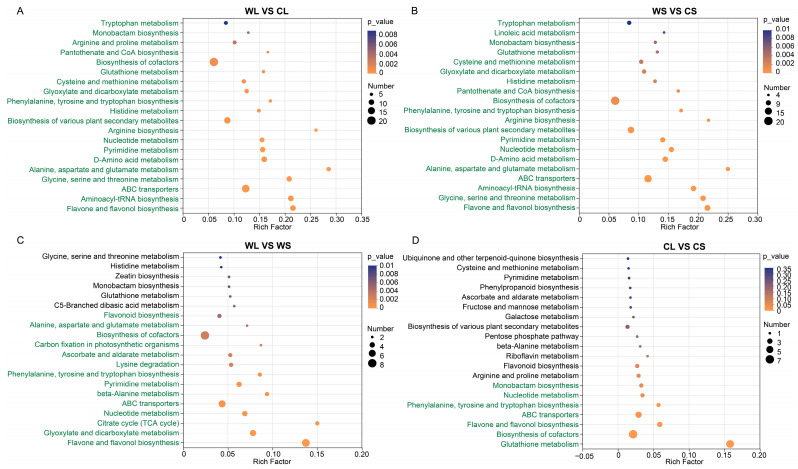
Functional pathway enrichment analysis of differential metabolites in different sample groups of wild and cultivated *Anoectochilus roxburghii*. (**A**) Functional pathway enrichment of wild leaves vs. cultivated leaves (WL vs. CL); (**B**) functional pathway enrichment of wild stems vs. cultivated stems (WS vs. CS); (**C**) functional pathway enrichment of wild leaves vs. wild stems (WL vs. WS); (**D**) functional pathway enrichment of cultivated leaves vs. cultivated stems (CL vs. CS).

To further investigate metabolic processes associated with the identified differential metabolites, KEGG pathway enrichment analysis was performed. As shown in Figure 3, pathways highlighted in green represent those that reached statistical significance (*p* < 0.05). In the comparison between wild and cultivated leaves (WL vs. CL), differential metabolites were primarily enriched in flavone and flavonol biosynthesis, aminoacyl-tRNA biosynthesis, ABC transporters, glycine, serine and threonine metabolism, and alanine, aspartate and glutamate metabolism (Figure 3A). A similar enrichment pattern was observed in the comparison between wild and cultivated stems (WS vs. CS), with largely overlapping significantly enriched pathways (Figure 3B).

In the comparison of wild plant tissues (WL vs. WS), differential metabolites were mainly enriched in flavone and flavonol biosynthesis, glyoxylate and dicarboxylate metabolism, the citrate cycle (TCA cycle), nucleotide metabolism, and ABC transporters (Figure 3C). In contrast, in the comparison of cultivated plant tissues (CL vs. CS), significantly enriched pathways included glutathione metabolism, biosynthesis of cofactors, flavone and flavonol biosynthesis, ABC transporters, phenylalanine, and tyrosine and tryptophan biosynthesis (Figure 3D).

Overall, several pathways were consistently enriched across comparisons, including flavonoid biosynthesis, ABC transporters, and multiple amino acid metabolism pathways. These results suggest that these metabolic processes contribute to both habitat-associated and tissue-specific metabolic differentiation in *A. roxburghii*.

### 3.4. Transcriptome Profiling and Differential Gene Expression in A. roxburghii

To examine transcriptional differences between wild and cultivated *A. roxburghii* across tissues, transcriptome sequencing was performed on 12 samples. After quality filtering, a total of 80.35 GB of clean data was obtained, with Q30 values exceeding 96.29% for all samples. Following redundancy removal, 157,514 unigenes and 276,186 transcripts were identified, indicating high sequencing depth and assembly quality and providing a robust basis for subsequent differential expression analysis and functional annotation.

**Figure 4:**
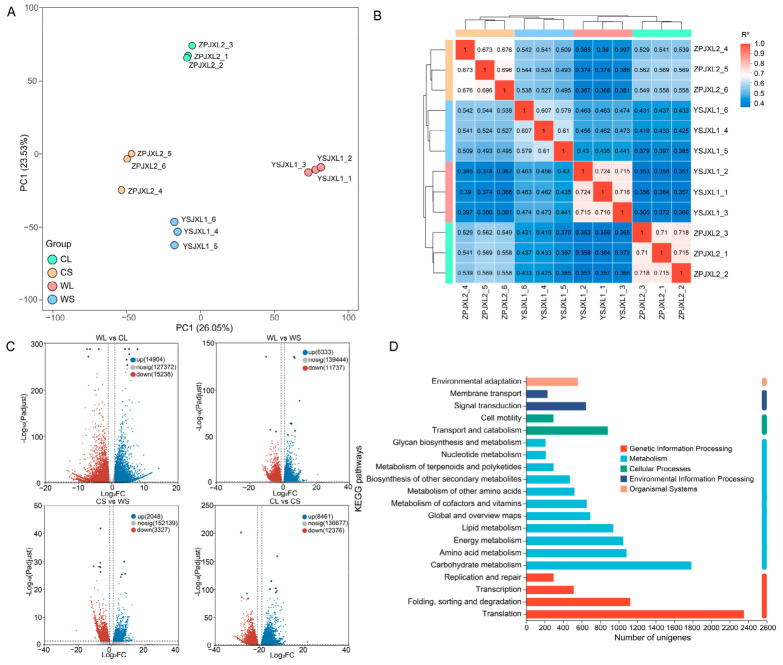
Differentially expressed gene analysis of *Anoectochilus roxburghii*. (**A**) Principal component analysis of transcriptomic gene expression profiles; (**B**) correlation heatmap showing relationships among different sample groups; (**C**) volcano plots of DEGs; (**D**) KEGG pathway classification of transcriptome.

PCA revealed clear separation among the four sample groups, with biological replicates clustering closely together (Figure 4A), indicating strong reproducibility and distinct transcriptional differences between groups. Wild and cultivated samples showed clear separation along the PC1 and PC2 axes, reflecting substantial variation in global gene expression patterns. This clustering pattern was further supported by the sample correlation heatmap (Figure 4B), which showed high correlation coefficients within groups and relatively lower correlations between groups.

Volcano plot analysis of DEGs (Figure 4C) revealed extensive transcriptional variation across both habitat- and tissue-based comparisons (Figure 4C). In the comparison between wild and cultivated leaves (WL vs. CL), 30,142 DEGs were identified, including 14,904 upregulated and 15,238 downregulated genes. In the comparison of stems (WS vs. CS), 5375 DEGs were detected, comprising 2049 upregulated and 3327 downregulated genes. In the wild plants, comparison between leaves and stems (WL vs. WS) identified 18,070 DEGs, including 6333 upregulated and 11,737 downregulated genes. Similarly, 20,837 DEGs were detected between cultivated leaves and stems (CL vs. CS), including 8461 upregulated and 12,376 downregulated genes.

Functional annotation based on KEGG classification indicated that the assembled unigenes were mainly distributed across several functional categories, including metabolism, genetic information processing, cellular processes, and environmental information processing (Figure 4D). Among these categories, metabolism-related pathways accounted for the largest proportion, primarily involving carbohydrate metabolism, amino acid metabolism, energy metabolism, and lipid metabolism. These results suggest that the *A. roxburghii* transcriptome is strongly associated with biosynthetic and energy conversion processes.

### 3.5. Functional Enrichment of Differentially Expressed Genes in A. roxburghii

**Figure 5:**
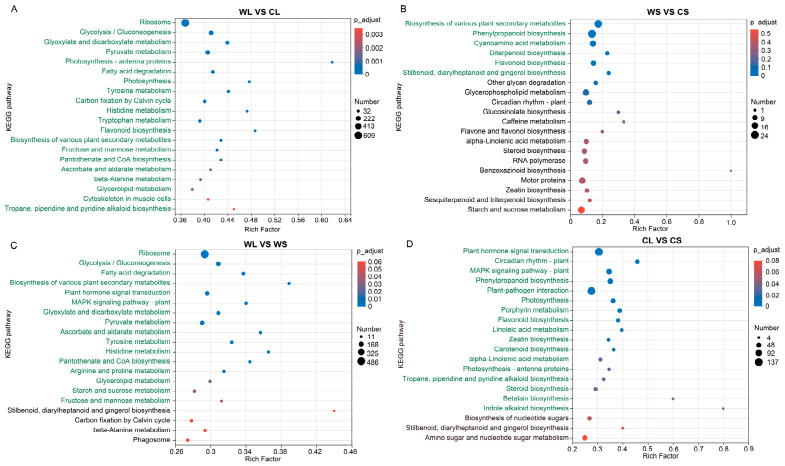
Functional pathway enrichment analysis of differentially expressed genes in different sample groups of *Anoectochilus roxburghii*. Functional pathway enrichment in comparison of (**A**) wild leaves vs. cultivated leaves (WL vs. CL); (**B**) wild stems vs. cultivated stems (WS vs. CS); (**C**) wild leaves vs. wild stems (WL vs. WS); and (**D**) cultivated leaves vs. cultivated stems (CL vs. CS).

DEGs between habitats and tissues were further examined to identify major functional patterns in the transcriptome. KEGG annotation was used to summarize the biological processes associated with these genes (Figure 5), with significantly enriched pathways indicated in green (*p* < 0.05).

In the comparison between wild and cultivated leaves (WL vs. CL), enriched pathways included ribosome, glycolysis/gluconeogenesis, fatty acid degradation, flavonoid biosynthesis, tropane, piperidine and pyridine alkaloid biosynthesis, ascorbate and aldarate metabolism, and photosynthesis (Figure 5A). In the comparison of stems (WS vs. CS), enriched pathways were mainly related to secondary metabolism, including biosynthesis of various plant secondary metabolites, phenylpropanoid biosynthesis, cyanoamino acid metabolism, diterpenoid biosynthesis, flavonoid biosynthesis, and stilbenoid, diarylheptanoid and gingerol biosynthesis (Figure 5B).

Tissue comparisons of plants from the same habitat revealed additional transcriptional differences. In the comparison of wild plant tissues (WL vs. WS), DEGs were enriched in ribosome, glycolysis/gluconeogenesis, fatty acid degradation, biosynthesis of various plant secondary metabolites, and plant hormone signal transduction (Figure 5C). In the comparison of cultivated plant tissues (CL vs. CS), enriched pathways included plant hormone signal transduction, circadian rhythm—plant, MAPK signaling pathway—plant, phenylpropanoid biosynthesis, plant–pathogen interaction, and photosynthesis (Figure 5D).

Overall, these results suggest that transcriptional differences between habitats and tissues are associated with variation in pathways related to both primary metabolic pathways and plant secondary metabolism in *A. roxburghii*.

### 3.6. Integrated Analysis of the Transcriptome and Metabolome in A. roxburghii

After adjustment for multiple testing (padjust < 0.05), transcriptomic and metabolomic data were examined jointly at the pathway level. In leaf tissues, analysis of both datasets identified 27 significantly enriched pathways, of which nine showed overlap between datasets. These pathways included alanine, aspartate, and glutamate metabolism; arginine and proline metabolism; β-alanine metabolism; glyoxylate and dicarboxylate metabolism; histidine metabolism; lysine degradation; pantothenate and CoA biosynthesis; tryptophan metabolism; and biosynthesis of plant secondary metabolites, including flavonoid biosynthesis. In the comparison of stem tissues from wild and cultivated plants, 24 enriched pathways were identified in the metabolomic dataset, whereas six were identified in the transcriptomic dataset. Among these, biosynthesis of various plant secondary metabolites was the only pathway significantly enriched in both datasets. In the leaf–stem comparison within the wild plant group, eight enriched pathways were detected at the metabolite level and 21 at the transcript level, with β-alanine metabolism and glyoxylate and dicarboxylate metabolism overlapping between datasets. In the cultivated plant group, four enriched pathways were identified in the metabolome and 18 in the transcriptome, with no overlapping pathways. Despite this limited overlap, both datasets showed involvement of pathways related to flavonoid metabolism, terpenoid metabolism, cofactor biosynthesis, and aromatic amino acid and phenylpropanoid metabolism.

**Figure 6:**
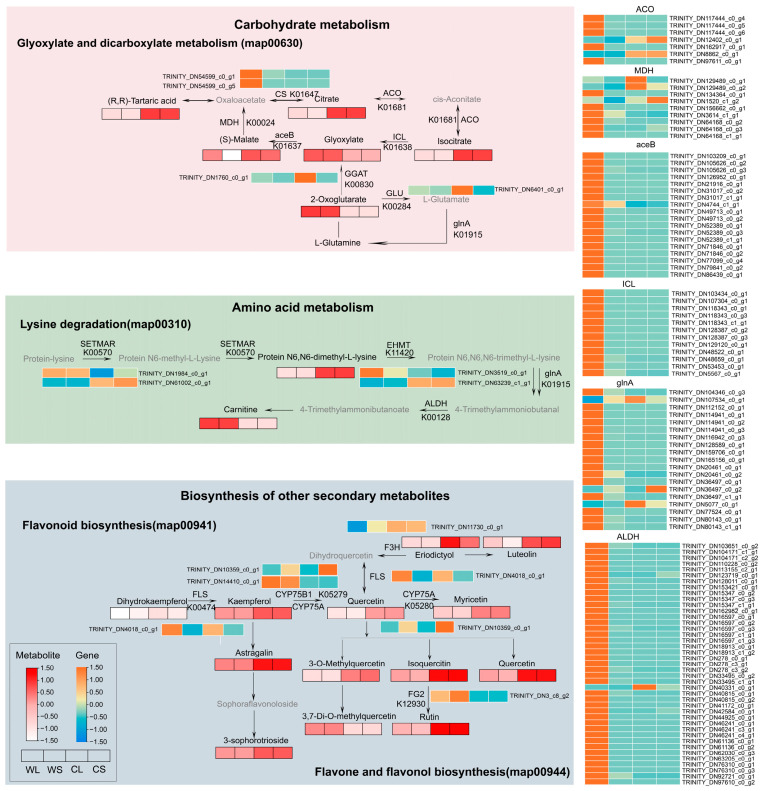
Integrated KEGG pathway analysis across different sample groups of *Anoectochilus roxburghii*. Combined transcriptomic and metabolomic data were mapped onto representative KEGG pathways, including glyoxylate and dicarboxylate metabolism (map00630), lysine degradation (map00310), flavonoid biosynthesis (map00941), and flavone and flavonol biosynthesis (map00944). Rectangles represent metabolites, and adjacent heatmaps indicate the expression levels of the corresponding structural genes across the four groups (WL, WS, CL, and CS). Color gradients reflect relative abundance or expression levels, with orange indicating higher levels and green indicating lower levels. Gray elements represent genes or metabolites that were detected but did not show significant differential changes across the compared groups.

Integrated analysis indicated concordant differences in metabolite abundance and gene expression associated with the glyoxylate and dicarboxylate pathway between wild and cultivated *A. roxburghii* (Figure 6). In wild plants, metabolites such as 2-oxoglutarate and glyoxylate showed higher abundance, and genes encoding enzymes involved in this pathway, including *MDH*, *ICL*, and *aceB*, showed higher expression in wild leaves. The nitrogen assimilation gene *glnA* exhibited a similar expression pattern. In contrast, several related metabolites, including (S)-malate, (R,R)-tartaric acid, citric acid, and isocitrate, accumulated at higher levels in cultivated plants. Differences were also observed in amino acid-related pathways. In the lysine pathway, the intermediate metabolite N6,N6-dimethyl-L-lysine accumulated to higher levels in cultivated plants, whereas the downstream product carnitine was more abundant in wild plants. Consistent with this pattern, *ALDH* genes involved in downstream oxidation steps showed higher expression in wild leaves.

Differences between habitats were particularly evident in flavonoid-related metabolites. Although relatively few structural genes were annotated in this pathway, metabolite-level changes were pronounced. Most flavonoid metabolites showed higher abundance in cultivated *A. roxburghii* plants. The gene *F3H* was significantly upregulated in cultivated plants and was associated with increased levels of eriodictyol and luteolin. Core flavonoid compounds, including kaempferol, quercetin, and myricetin, as well as their glycosylated derivatives astragalin, rutin, isoquercitrin, and kaempferol 3-sophorotrioside, also showed higher accumulation in cultivated plants.

### 3.7. Correlation Analysis of Flavonoid Metabolites and Associated Genes in A. roxburghii

**Figure 7:**
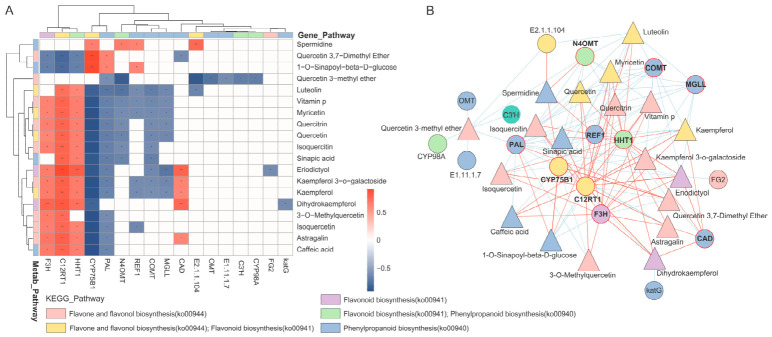
Association analysis of genes and metabolites involved in flavonoid and phenylpropanoid biosynthesis pathways. (**A**) Gene–metabolite correlation heatmap; (**B**) gene–metabolite interaction network. Triangular nodes represent metabolites, and circular nodes represent genes, while node colors correspond to their KEGG pathway classifications. Red edges indicate significant positive correlations, whereas blue edges indicate significant negative correlations. Red circular nodes denote hub genes that contribute strongly to network connectivity or are associated with central positions in the pathway network.

To examine associations between flavonoid metabolites and gene expression, Pearson correlation analysis (|*r*| > 0.6, *p* < 0.05) was performed to construct gene–metabolite correlation heatmaps and networks (Figure 7). The heatmap analysis (Figure 7A) showed that several flavonoid metabolites, including luteolin, quercetin, quercitrin, and kaempferol, were positively correlated with structural genes such as *F3H*, *C12RT1*, and *HHT1*. In contrast, these metabolites showed negative correlations with genes such as *CYP75B1* and *COMT*. Network analysis (Figure 7B) further illustrated multiple connections between flavonoid metabolites and genes associated with related biosynthetic pathways. Genes such as *CYP75B1*, *C12RT1*, *PAL*, *HHT1*, and *F3H* showed relatively high connectivity within the correlation network.

**Figure 8:**
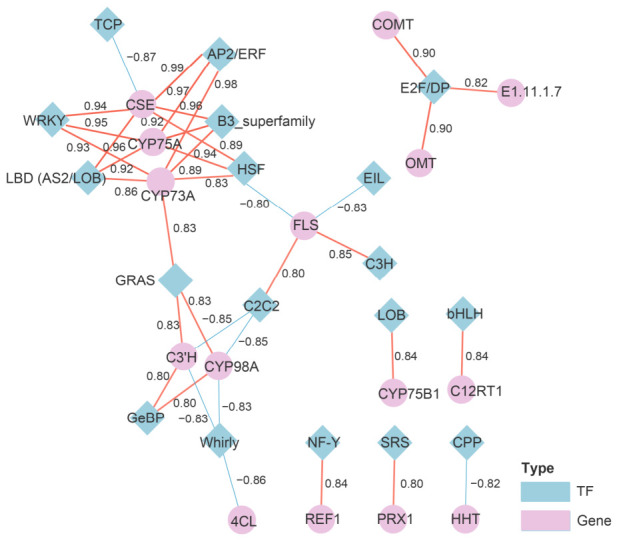
Correlation network between TFs and key structural genes involved in the flavonoid biosynthesis pathway in *Anoectochilus roxburghii*.

TFs were further examined to explore potential associations with flavonoid-related genes. Transcriptome analysis identified 853 TF genes belonging to 32 TF families. The ten most abundant families were MYB (133), AP2/ERF (79), C2C2 (67), NAC (62), FAR1 (54), GRAS (54), bHLH (49), bZIP (45), B3 (35), and WRKY (35). Subsequently, Pearson correlation analysis (|*r*| > 0.8, *p* < 0.05) was performed to construct a TF–gene correlation network (Figure 8). The network showed both positive and negative correlations between TFs and flavonoid-related genes. Several TF families, including AP2/ERF, B3, HSF, WRKY, and LBD (AS2/LOB), showed positive correlations with genes such as *CSE*, *CYP75A*, and *CYP73A*. In contrast, some TFs showed negative correlations with flavonoid-related genes; for example, *TCP* was negatively correlated with *CSE*, and Whirly showed negative correlations with *CYP98A* and *C3′H*.

## 4. Discussion

### 4.1. Habitat-Associated Metabolic Differences in A. roxburghii

Integrated metabolomic analysis revealed clear differences in metabolite profiles between wild and cultivated *Anoectochilus roxburghii*. Wild plants exhibited higher levels of several amino acids and nitrogen-containing metabolites, including Arg–Ser–Asn and 4′-thiothymidine, together with enrichment of metabolites mapped to the glyoxylate and dicarboxylate pathway. These metabolites and pathways are related to fundamental processes of carbon and nitrogen metabolism. In natural forest habitats, A. roxburghii experiences variable environmental conditions, including fluctuating light availability, heterogeneous soil nutrients, and complex rhizosphere microbial communities. Such environmental variability may be associated with metabolic processes related to carbon and nitrogen metabolism [28,29]. In line with this context, in this study, wild plants showed higher levels of several amino acids and nitrogen-containing metabolites, as well as enrichment of metabolites associated with the glyoxylate and dicarboxylate pathway.

By contrast, cultivated plants are typically grown under more controlled conditions, including relatively stable shading and substrate composition. Under these conditions, cultivated tissues were characterized by higher levels of several flavonoids and phenolic compounds, including isoquercetin, robinetin, dihydromyricetin 3-rhamnoside, and 7-O-prenylscopoletin [30,31,32]. Similar patterns of increased flavonoid accumulation under cultivation conditions have been reported in other Orchidaceae species and medicinal plants [33,34]. Taken together, these results suggest an association between habitat conditions and differences in metabolite composition between wild and cultivated *A. roxburghii*, particularly in the relative abundance of primary metabolic compounds and flavonoid-related secondary metabolites.

### 4.2. Tissue-Specific Metabolic Differentiation Between Leaves and Stems

In this study, leaves and stems exhibited clear metabolic differentiation, consistent with their distinct physiological roles in *A. roxburghii*. Leaves act as the primary photosynthetic organs and are directly exposed to fluctuating light and atmospheric conditions, consistent with active primary metabolism related to carbon assimilation and energy production [35]. In contrast, stems serve mainly structural and transport functions, facilitating nutrient allocation and supporting vegetative growth [36].

Consistent with these functional differences, several flavonoid glycosides show higher accumulation in stems than in leaves [37,38,39]. In many perennial medicinal plants, stems can serve as important sites for the accumulation of bioactive secondary metabolites, which have been associated with structural protection and stress tolerance [40]. The enrichment of specific flavonoids in stems observed in this study may therefore be related to tissue-specific metabolite partitioning associated with distinct physiological functions, rather than habitat effects alone [32,41]. Notably, tissue-level metabolic patterns differed between wild and cultivated plants, indicating that the magnitude and direction of organ-specific metabolite variation varied under different growth conditions. These observations suggest that both tissue identity and habitat conditions are associated with variation in the metabolic profiles of *A. roxburghii* [42].

### 4.3. Primary Metabolic Pathways Associated with Secondary Metabolite Biosynthesis

Primary metabolic pathways supply precursors required for the biosynthesis of many secondary metabolites, including flavonoids [43,44,45]. In wild *A. roxburghii*, higher levels of glyoxylate and 2-oxoglutarate were detected, together with increased expression of several genes involved in carbon and nitrogen metabolism, including *ICL*, *MDH*, *aceB*, and the nitrogen assimilation gene *glnA*. However, several related organic acid intermediates, including malate, citrate, and isocitrate, accumulated at higher levels in cultivated plants. This apparent discordance between gene expression and metabolite accumulation patterns suggests that elevated transcript levels do not necessarily correspond to increased metabolite flux in a straightforward manner, as metabolite accumulation is influenced by multiple factors, including downstream consumption, transport, and competing metabolic pathways. Elevated levels of carnitine and increased expression of *ALDH* genes were also observed in wild samples. These patterns are consistent with differences in primary metabolic processes between wild and cultivated plants. Further experimental validation would be required to clarify the relationships between gene expression levels and metabolite accumulation in the pathway underlying these patterns.

In cultivated plants, relatively higher levels of several organic acid intermediates were observed, including malate, citrate, and isocitrate, as well as modified lysine derivatives such as N6,N6-dimethyl-L-lysine [46]. These metabolites are commonly associated with central carbon and amino acid metabolism. Differences in the abundance of these primary metabolites may be associated with the availability of metabolic intermediates linked to secondary metabolite biosynthesis, including the phenylpropanoid and flavonoid pathways. Taken together, the integrated metabolomic and transcriptomic results suggest that variation in primary metabolic pathways is associated with differences in secondary metabolite accumulation between wild and cultivated *A. roxburghii* [47].

### 4.4. Gene Expression Patterns Associated with Flavonoid Accumulation

Transcriptomic analysis revealed differential expression of several genes involved in flavonoid biosynthesis between wild and cultivated *Anoectochilus roxburghii*. Among the annotated structural genes, *F3H* was significantly upregulated in cultivated plants, corresponding with higher levels of several flavonoid metabolites, including eriodictyol, luteolin, and related flavonol derivatives. *F3H* is widely recognized as a key enzyme linking phenylpropanoid metabolism to flavonoid biosynthesis [48]. Gene–metabolite correlation analysis further indicated that multiple flavonoid metabolites were positively correlated with *F3H*, *C12RT1*, and *HHT1*, and negatively correlated with *CYP75B1* and *COMT* [49]. These patterns suggest that variation in flavonoid accumulation may be associated with coordinated expression of multiple biosynthetic genes rather than variation in a single gene. The presence of highly connected nodes in the correlation network also indicates potential relationships among genes and metabolites within the flavonoid pathway. In addition, transcription factor analysis identified associations between members of the MYB, bHLH, WRKY, and AP2/ERF families and flavonoid-related structural genes [50,51]. These TF families are widely reported to participate in the regulation of flavonoid biosynthesis in many plant species, including members of *Orchidaceae*. In this study, their expression patterns were correlated with several flavonoid biosynthetic genes, suggesting potential involvement in flavonoid-related metabolic processes.

### 4.5. Limitations

Several limitations of this study should be acknowledged. First, in the absence of genotyping data, we cannot fully disentangle the contribution of genetic background and habitat conditions to the observed transcriptomic and metabolic differences. Wild and cultivated populations might have undergone genetic divergence through natural selection or artificial selection during domestication, and the extent to which the metabolic variation reported herein reflects heritable genetic differences versus plastic responses to environmental conditions remains unclear. Future studies employing common garden experiments or population-level genetic analyses (e.g., SNP-based genotyping) would be necessary to resolve this issue. Second, although the two collection sites share similar geographic, climatic, and edaphic characteristics, quantitative environmental monitoring data—such as continuous records of light intensity, soil nutrient profiles, pH, moisture content, and microbial community composition—were not collected in this study. As a result, we cannot precisely attribute the observed metabolic differences to specific environmental variables. Third, all gene–metabolite and transcription factor–gene associations reported in this study are based on correlation analyses rather than experimental validation. Correlation does not establish causation, and the regulatory relationships inferred from network analyses should be regarded as hypotheses requiring functional confirmation through approaches such as gene silencing, overexpression, or enzyme activity assays.

## 5. Conclusions

Taken together, the comparative metabolomic and transcriptomic analyses revealed clear differences in metabolite profiles and gene expression patterns between wild and cultivated *Anoectochilus roxburghii*, as well as between leaves and stems. Wild plants exhibited higher levels of several amino acids and primary metabolic intermediates, whereas cultivated plants accumulated higher levels of multiple flavonoids and phenolic compounds. In addition, metabolite profiles differed between leaves and stems, consistent with the tissue-specific metabolic characteristics associated with their distinct physiological roles. Overall, these findings suggest that both habitat conditions and tissue identity are associated with variations in primary and secondary metabolic profiles in *A. roxburghii*. Further experimental studies will be required to clarify the underlying biological mechanisms.

## Data Availability

The datasets presented in this study can be found in the National Genomics Data Center (NGDC), with the accession number PRJCA054161.

## Supplementary Materials

The following supporting information can be downloaded at https://www.mdpi.com/article/10.3390/metabo16050294/s1. Figure S1: Classification and distribution of identified metabolites in *Anoectochilus roxburghii*; Figure S2: Differential metabolites identified in pairwise comparisons of *Anoectochilus roxburghii*; Table S1: Summary of sample collection details for wild and cultivated *Anoectochilus roxburghii*.
