# Supplementary material for: Integrated Metabolomics and Transcriptomics Reveal the Influence of Natural and Cultivation-Managed Habitats on Metabolic Divergence and Flavonoid Enrichment in Anoectochilus roxburghii
Source: Metabolites. 2026 Apr 27;16(5):294. doi: 10.3390/metabo16050294 (PMC13209090; doi:10.3390/metabo16050294)
Supplement: Supplementary file 1 [file metabolites-16-00294-s001.zip › metabolites-4176964-supplementary.pdf]

Supplementary Information for

Integrated Metabolomics and Transcriptomics Reveal the Influence of Natural and Cultivation-Managed Habitats on Metabolic Divergence and Flavonoid Enrichment in *Anoectochilus roxburghii*

Authors: Erli Wang[1], Weicheng Gao[1], Peng Wang[2] and Xiaoping Wang[1*]

Author affiliations:

[1]School of Pharmacy, Zhangzhou Health Vocational College, Zhangzhou 363000, China

[2]Zhiran Biotechnology Co., Ltd., Tianjin 301000, China

[*]Correspondence: wangxp@zzwzy.edu.cn

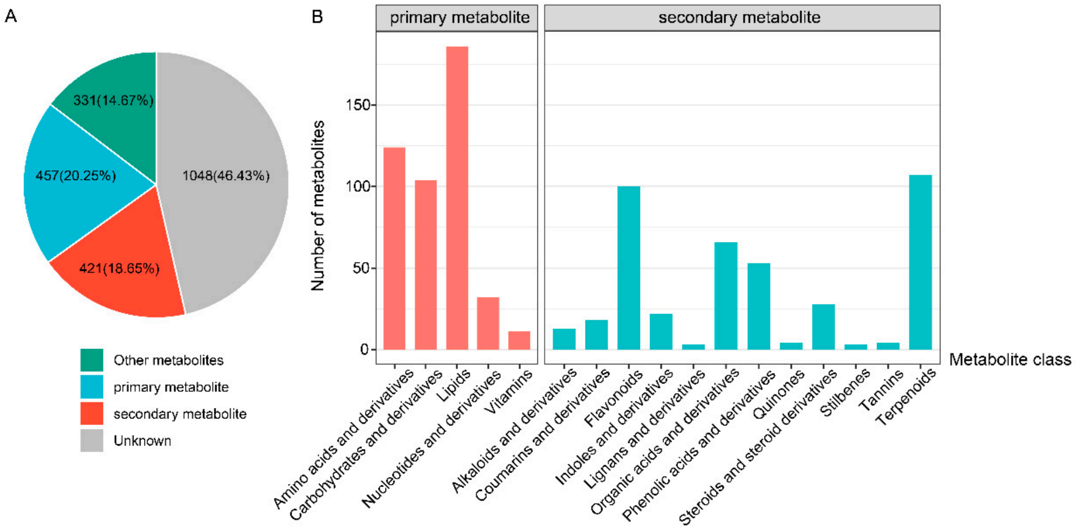

Figure S1. Classification and distribution of identified metabolites in *Anoectochilus roxburghii*. (A) Pie chart showing the overall composition of detected metabolites, categorized into primary metabolites, secondary metabolites, other metabolites, and unclassified compounds, with corresponding proportions indicated. (B) Bar plots showing the number of metabolites within each subclass, grouped into primary metabolites (left panel) and secondary metabolites (right panel). Primary metabolites are mainly composed of amino acids and derivatives, carbohydrates and derivatives, lipids, nucleotides and derivatives, and vitamins. Secondary metabolites include flavonoids, phenolic acids and derivatives, terpenoids, alkaloids and derivatives, and other minor classes. The y-axis represents the number of metabolites in each category.

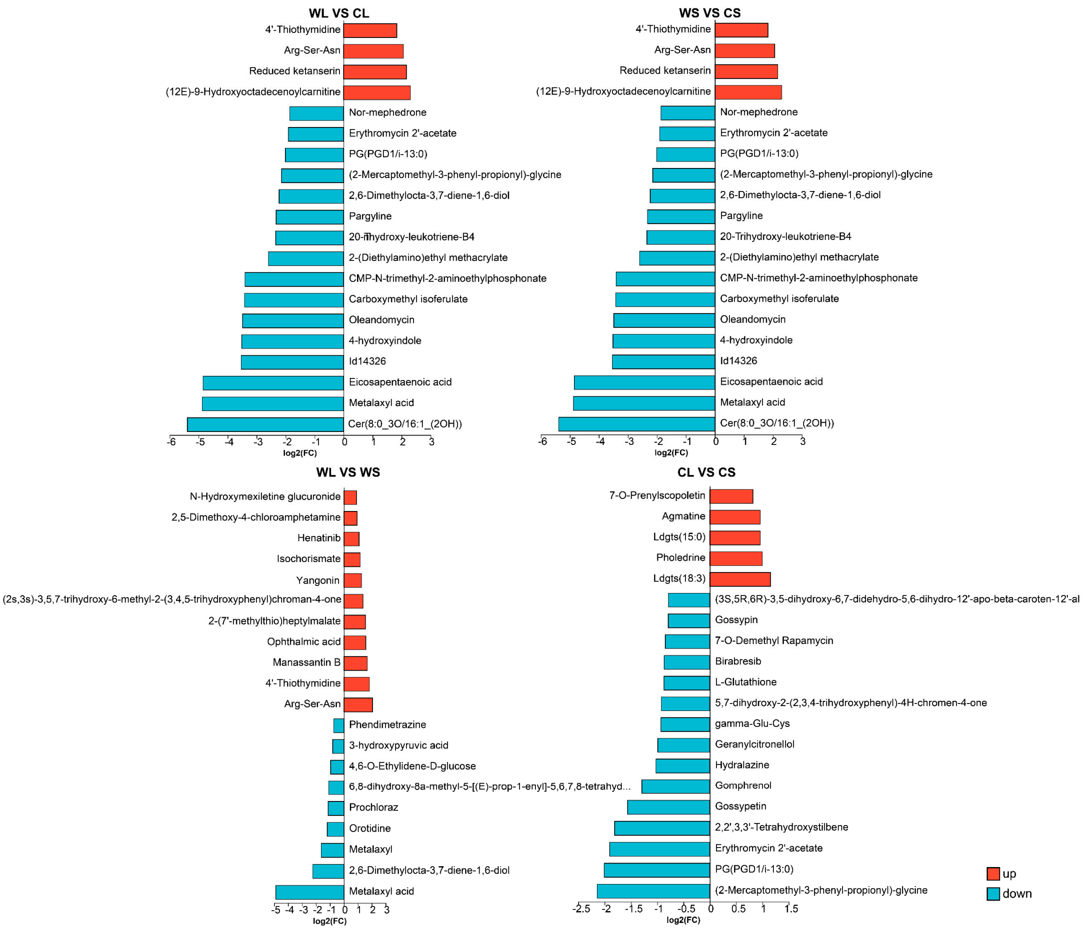

Figure S2. Differential metabolites identified in pairwise comparisons of *Anoectochilus roxburghii*. Bar plots showing representative differential metabolites in four comparisons (WL vs. CL, WS vs. CS, WL vs. WS, and CL vs. CS). The x-axis indicates log2 fold change [log2(FC)]. Red bars represent metabolites enriched in the first group of each comparison, while blue bars represent those enriched in the second group.

Table S1. Summary of sample collection details for wild and cultivated *Anoectochilus roxburghii.*

Sample Code	Collection Type	Habitat Location	Plant Part	Biological Replicates	Individuals per Replicate	Collection Site
WL	Wild	Natural forest understory	Leaf	3	7	Shajian Town, Hua'an County (24°47′N, 117°05′E; 1,020 m)
WS	Wild	Natural forest understory	Stem	3	7	Shajian Town, Hua'an County (24°47′N, 117°05′E; 1,020 m)
CL	Cultivated	Forest-understory semi-wild cultivation	Leaf	3	10	Shuyang Town, Nanjing County (24°35′N, 117°05′E; 1,052 m)
CS	Cultivated	Forest-understory semi-wild cultivation	Stem	3	10	Shuyang Town, Nanjing County (24°35′N, 117°05′E; 1,052 m)

Notes: All samples were collected during mid-October (09:00–11:00) from Zhangzhou, Fujian Province, China. Plants were selected based on uniform morphological criteria: plant height ⩾12 cm, 5–6 fully expanded leaves, well-developed root systems, and no visible disease or pest damage. Cultivated plants had been grown under forest-understory conditions for approximately 15 months. Multiple individuals were pooled per biological replicate to minimize individual-level variability and better represent population-level metabolic profiles. Fresh tissues were immediately frozen in liquid nitrogen within 5 min of harvesting and stored at −80 °C.
